# The epidemiology and transmission of methicillin-resistant *Staphylococcus aureus* in the community in Singapore: study protocol for a longitudinal household study

**DOI:** 10.1186/s12879-017-2793-y

**Published:** 2017-10-11

**Authors:** Nivedita Shankar, Angela Li Ping Chow, Jolene Oon, Li Yang Hsu, Brenda Ang, Junxiong Pang, Paola Florez De Sessions, Balamurugan Periaswamy, Paul A. Tambyah, Desmond B. Teo, Clarence C. Tam

**Affiliations:** 1 Saw Swee Hock School of Public Health, Tahir Foundation Building, 12 Science Drive 2, 11-01, Singapore, 117549 Singapore; 2 Tan Tock Seng Hospital, Singapore, Singapore; 3 National University Hospital, Singapore, Singapore; 4 Genome Institute of Singapore, Singapore, Singapore; 5 London School of Hygiene and Tropical Medicine, London, UK

**Keywords:** Cohort studies, Longitudinal studies, MRSA, *Staphylococcus aureus*, Methicillin, Antimicrobial resistance epidemiology, Community, Colonization, Household transmission

## Abstract

**Background/aim:**

Methicillin-resistant *Staphylococcus aureus* (MRSA) is one of the most common multidrug-resistant organisms in healthcare settings worldwide, but little is known about MRSA transmission outside of acute healthcare settings especially in Asia. We describe the methods for a prospective longitudinal study of MRSA prevalence and transmission.

**Methods:**

MRSA-colonized individuals were identified from MRSA admission screening at two tertiary hospitals and recruited together with their household contacts. Participants submitted self-collected nasal, axilla and groin (NAG) swabs by mail for MRSA culture at baseline and monthly thereafter for 6 months. A comparison group of households of MRSA-negative patients provided swab samples at one time point. In a validation sub-study, separate swabs from each site were collected from randomly selected individuals, to compare MRSA detection rates between swab sites, and between samples collected by participants versus those collected by trained research staff. Information on each participant’s demographic information, medical status and medical history, past healthcare facilities usage and contacts, and personal interactions with others were collected using a self-administered questionnaire.

**Discussion/conclusion:**

Understanding the dynamics of MRSA persistence and transmission in the community is crucial to devising and evaluating successful MRSA control strategies. Close contact with MRSA colonized patients may to be important for MRSA persistence in the community; evidence from this study on the extent of community MRSA could inform the development of household- or community-based interventions to reduce MRSA colonization of close contacts and subsequent re-introduction of MRSA into healthcare settings. Analysis of longitudinal data using whole-genome sequencing will yield further information regarding MRSA transmission within households, with significant implications for MRSA infection control outside acute hospital settings.

## Background

Since its emergence in the 1960s, methicillin-resistant *Staphylococcus aureus* (MRSA) has become one of the most common multidrug-resistant organisms in healthcare settings worldwide, resulting in considerable health and economic burden. Carriage of MRSA is a major risk factor for invasive MRSA infection [1–8], and patient transfers between acute care hospitals, community hospitals and long-term care facilities have been reported to be important for maintaining MRSA transmission in healthcare settings [9–17]. MRSA transmission from MRSA-infected patients and MRSA carriers to their household contacts has also been documented [18–22], but the importance of household transmission for persistence of MRSA in the community and reintroduction into healthcare settings is not well understood. A number of cross-sectional studies have suggested that between 20% and 30% of household contacts of MRSA positive index patients are colonized with MRSA [23, 24]. A limited number of longitudinal studies have shown that MRSA transmission to household contacts is common, with 20% - 40% of non-colonized household contacts of MRSA positive individuals acquiring MRSA during the follow-up period [18, 20–22]. Identified risk factors for MRSA acquisition among household contacts include older age [20] and providing care to MRSA-colonized individuals [20, 25].

In Singapore, MRSA is endemic in healthcare facilities [26, 27]. A laboratory-based program in six public sector acute care hospitals (ACHs) reported that 35% of all clinical *S. aureus* isolates were methicillin-resistant [26]. MRSA Sequence Type (ST) 239 was predominant in Singaporean hospitals in the mid-1980s, but was rapidly supplanted as the major clone following the introduction of ST22 in the early 2000s [28]. More recently, ST45 was introduced into local hospitals. Community-associated (CA) MRSA strains have also been identified sporadically since the early 2000s, with ST30 being the predominant clonal strain [29].

Specific measures adopted by healthcare institutions to control MRSA include active surveillance in tertiary care hospitals, cohorting or isolation of MRSA colonized patients and hand hygiene initiatives. Historical evaluations of these bundled MRSA interventions at two major tertiary care hospitals have documented a sustained decrease in MRSA infection and hospital acquired bacteraemia rates of 50% - 60% between 2004 and 2012, alongside significant increases in hand hygiene compliance among healthcare workers [30]. Reducing colonization is believed to be crucial to reducing MRSA burden, as colonization is a major risk factor for subsequent infection, and infection carries a high risk of excess morbidity and mortality [25, 26, 31, 32].

Despite these efforts to control MRSA transmission in ACHs, intermediate-term and long-term care facilities remain as important reservoirs [11]. A recent survey indicated that MRSA colonization prevalence is substantially higher among patients in intermediate-term (29.9%) and long-term (20.4%) care facilities compared with acute care hospitals (11.8%) [11]. Several studies have suggested a role of long-term care facilities for sustaining MRSA endemicity in healthcare networks [11, 14, 16, 17, 33]. However, relatively little is known about the role of MRSA transmission in the wider, non-hospitalized community in maintaining MRSA transmission and reintroducing MRSA strains into healthcare settings. A few longitudinal studies have demonstrated the potential for MRSA transmission from colonized patients to their household contacts, suggesting the existence of a large reservoir of MRSA in the general population. However, the relationship between these community transmission events and the epidemiology of MRSA in healthcare settings is not well understood.

In recent years, whole genome sequencing (WGS) has emerged as the leading technology for the study of bacterial population genetics and transmission dynamics [34–37]. WGS has provided crucial insights into the epidemiology of antibiotic resistance, the genetics of virulence, the origins and expansion of lineages, and the population structure of *S. aureus* [38]. The high resolution of WGS means that it offers superior data compared with other molecular typing techniques to determine transmission events, and profile resistance, virulence and even the type isolates [38–40] within days [41]. Various studies have used WGS to track MRSA transmission networks in healthcare settings [9, 11, 12, 33, 42–44] and the community [44]. To our knowledge, however, no study has investigated MRSA carriage and transmission among households using WGS. We describe the methods for an ongoing longitudinal study to investigate the persistence and transmissibility of MRSA in the community, and to quantify the relationship between healthcare contact and community transmission.

## Methods

The overall aim of this study is to determine the dynamics of MRSA carriage, persistence and transmission in the community. The specific objectives are:

- To measure frequency of MRSA colonization among household contacts
- To identify risk factors associated with colonization and prolonged carriage
- To study MRSA transmission in household contacts of MRSA-colonized patients (after discharge)
- To identify factors associated with MRSA persistence in the household
- To quantify importance of healthcare contact, and other personal interactions, in the transmission of MRSA in the community
- To determine the diversity of MRSA strains

This study will comprise a baseline survey of MRSA prevalence and risk factors among household contacts of 100 MRSA-colonized and 100 MRSA non-colonized index patients, followed by a 6-month longitudinal study among MRSA-colonized index patients and their household contacts, with monthly self-swabbing and questionnaire interview.

Index patients will be recruited from two acute and tertiary care hospitals in Singapore. Hospital 1 has 1600 beds and primarily serves an elderly population. Active MRSA admission screening based on nasal swab polymerase chain reaction (PCR) has been conducted since 2009. Positive patients are cohorted in dedicated MRSA wards. Hospital 2 is a university hospital with 1000 beds serving a mixed population. Active MRSA admission screening based on nose, axilla and groin swab culture has been conducted since 2010. Positive patients are allocated to dedicated MRSA cubicles within each ward.

Potential index patients for the MRSA-colonized and MRSA non-colonized arms will be selected from daily lists of patients undergoing MRSA screening on admission. Index patients will be eligible for inclusion in the MRSA-colonized arm if:

1. They are aged 21 years or above
2. They have a positive MRSA screen at admission
3. They live in a household with at least one other person who routinely spends nights at the same address

Patients will be excluded if they live in an institutionalized setting, such as a nursing home, or if they are admitted to an intensive care unit at the time of recruitment. Eligibility and exclusion criteria for index patients in the MRSA non-colonized arm will be the same, with the exception that the patient must test negative for MRSA upon admission screening.

Eligible patients will be approached in the ward by trained research staff to explain the study in the patients’ preferred language (English, Mandarin, Malay or Tamil) and to invite them to participate in the study. Interested patients will be asked to provide signed informed consent and consent to contact their household for the purposes of recruiting household members. For potential participants suffering from cognitive impairment, informed consent will be obtained from their legally appointed representative, usually a spouse or family member responsible for making healthcare decisions on behalf of their patients.

Research staff will then arrange a house visit to recruit household contacts of the index case. A household contact will be defined as a person who routinely spends nights at the same address as the index patient. Household contacts interested in taking part will be asked to provide signed informed consent. Parental consent and participant assent will be obtained for participants aged six to 20 years; children below six years will not be eligible to participate. A baseline questionnaire will be administered to each participating household member, to collect information on each participant’s demographic information, medical status and medical history, past healthcare facilities usage and contacts, and personal interactions with others. Participants will then be shown the correct procedure for obtaining swabs from the nares, axilla and groin (N, A, G), and asked to provide an N and combined AG swab using a Copan E-swab MRSA Collection System (Copan Diagnostics).

For participants in the MRSA-colonized arm taking part in the follow-up component, written instructions and links to instructional videos for the self-swabbing procedure will be provided for future reference. Participants will be instructed to take two swabs, one from the nares and a second from the axillary and groin areas, and combine them in a single sample tube. Colour-coded stickers will be pre-labelled with participants’ identification number and used to label the sample tubes. Pre-paid, addressed, padded envelopes will be provided for participants to send the swabs directly to the laboratory. Participants will submit swabs two and four weeks after enrolment and at monthly intervals thereafter, at a pre-specified time of the month, for a period of 6 months. Reminders will be sent by SMS. After 6 months, a second household visit will be arranged to update information on healthcare contacts in the previous 6 months, recent travel history and contact patterns.

In a validation sub-study, the research team will take swabs of the nares and axilla from a randomly selected household member at the initial recruitment visit. The selected household member will then take repeat swabs by themselves, and take a third swab from the groin area in a separate room. The five swab samples will be collected in separate sample tubes, using the Copan E-swab MRSA Collection System, and cultured individually to validate the self-swabbing procedure to be used for MRSA testing during the follow-up period.

Receipt of swab specimens will be recorded at the laboratory located near hospital 1. A study team member will liaise with the laboratory to ensure that specimens have been received. Households from which samples have not been received by the pre-specified date will be contacted by the research nurse to ensure that samples are taken and sent to the laboratory.

### Sample size

Given an average household size of three persons, we anticipate 200 household contacts in each arm. Lucet et al. reported 88% participation among household contacts^16^. Assuming 80% participation among household contacts, a study with 160 participants in each arm, for a total sample size of 320, will enable us to detect a minimum of 13% difference in MRSA prevalence between the two groups. These estimates assume a Type I error probability of 0.05, 80% power and a design effect (DEFF) of 1.25 resulting from the clustered nature of the survey design. We expect the design effect to be minimal, because of the small average number of participants recruited per household.

### Laboratory procedures

All swabs will be tested for MRSA by culture on selective chromogenic media (Bio-Rad MRSA *Select™),* followed by a confirmatory agglutination test (Bio-Rad Pastorex Staph Plus). If the first two tests are deemed inconclusive, a third coagulase test will be conducted.

Swab samples were plated onto selective chromogenic media using sterile inoculating loops.The plates will be incubated at 35-37 °C at ambient temperature and read at 18–24 h. Negative plates or plates with minimal growth will be re-incubated and re-read at 48 h. A latex agglutination test will be performed on culture plates with suspected MRSA colonies to confirm the presence of *Staphylococcus aureus*.

### Storage of MRSA isolates

Using a sterile loop, a mixture of different MRSA colonies will be picked and emulsified in the cryopreservative fluid. If culture growth is scarce, a subculture will be performed and the colonies picked for storage in cryocare vials (Key Scientific Products, Stamford, TX) at −80 °C.

### Whole genome sequencing of MRSA isolates

The whole genome sequencing phase of the study will require DNA to be extracted from the MRSA isolates. The MRSA isolates from the hospital laboratory are re-cultured on blood agar plates (BD Diagnostics, USA BBL™ Columbia Agar with 5% sheep blood) from cryocare vials, and 5 individual colonies are picked per plate for overnight culture in Luria-Bertani (LB) broth (Gibco® LB Broth, Liquid). 900 μl from each overnight culture is resuspended with 100 μl of sterile DMSO and stored as independent MRSA isolates. The remaining DNA will be extracted from these overnight cultures using the QIAamp DNA Mini Kit (Qiagen Inc., Hilden, Germany).

Where multiple transmissions are suspected between household members (for example, where a previously negative household member is subsequently colonized), a subset of strains from serial swabs will undergo whole genome sequencing for molecular epidemiological studies. Genomic DNA will be extracted from *S. aureus* cultures derived from swab samples of index patients and household members. The isolates will be used to provide background genetic information on the *S. aureus* strains circulating locally. Genomic DNA will be extracted using the QIAamp DNA Mini Kit (Qiagen Inc., Hilden, Germany). DNA shearing will be done using Covaris LE220 Focused-ultrasonicator followed by library preparation using NEBNext® Ultra™ DNA Library Prep Kit for Illumina® (E7370L) and custom primers with 8 bp barcode index. Sequencing will be done on an Illumina HiSeq 4 K platform.

## Ethical approval

Ethical approval for this study has been obtained from the National Health Group Domain Specific Review Board (NHG-DSRB 2014/01278).

### Data analysis

MRSA test results for duplicate swabs taken by trained researchers, considered a “gold-standard test”, and participants themselves from the same site will be used to determine the sensitivity of self-swabbing. Sensitivity will be defined as the proportion of positive nurse-administered swabs for which the paired, self-administered swab is also positive. Sensitivity will be calculated for MRSA, and separately for nasal and axillary swabs. Sensitivity estimates will be expressed as percentages with corresponding 95% confidence intervals (CI).

Duration of MRSA carriage, by anatomical site, will be quantified using survival analysis methods. Survival curves will be plotted to show the changes in carriage over time from serial samples. Survival curves will also be used to investigate the incidence of colonization among initially MRSA negative household contacts. The longitudinal nature of data from serial samples will be used to identify household and individual factors independently associated to MRSA colonization and time to MRSA clearance, using the Cox regression model. Associations between time to clearance and independent risk factors will be estimated by means of hazard ratios, with corresponding 95% CIs. Robust methods will be used to estimate standard errors, to account for dependence of observations within households.

Data on chains of transmission with clonal strains within households will enable estimation of household reproduction numbers, i.e. the average number of colonizations among household contacts arising from an index patient, as well as enabling assessment of MRSA colonization resulting from contact with sources other than household contacts.

Phylogenetic analysis of isolated strains will enable study of the genetic relatedness of MRSA strains associated with hospital encounters and MRSA strains circulating in the community.

## Discussion

As in many other countries, MRSA is endemic in Singapore. Increased efforts at infection control in hospitals in recent years have contributed to a reported decline in MRSA burden and subsequent stabilization of disease incidence [30].

Studies of household contacts of MRSA index patients have not been conducted in Singapore, and no longitudinal studies in the community have been done in Southeast Asia. This project is novel since it will be the first to conduct a longitudinal investigation of community transmission of MRSA in Singapore. The study will help determine the direction of transmission and the MRSA carriage dynamics over time. Understanding the dynamics of MRSA persistence and transmission in the community is crucial to devising and evaluating successful MRSA control strategies. Close contact with MRSA colonized patients may be important for MRSA persistence in the community; evidence from this study on the extent of community MRSA could inform the development of household- or community-based interventions to reduce MRSA colonization of close contacts and subsequent re-introduction of MRSA into healthcare settings.

The study will use whole genome sequencing (WGS) to delineate the molecular epidemiology of MRSA within the healthcare system in Singapore. This will help to determine whether these different types of institutions share a common epidemiology dictated by the continuing traffic of MRSA between institutions. MRSA isolates from nasal, axillary and groin (NAG) swab cultures will be typed by WGS and phylogenetic analysis used to establish the relationship between MRSA populations in these different settings. Data from WGS provides superior resolution and is less prone to false-positive and false-negative findings [45]. WGS makes it possible to achieve informative phylogenetic data which can provide additional information about transmission events. Phylogenetic analysis of isolated strains will also enable the study of the genetic relatedness of MRSA strains associated with hospital encounters and MRSA strains circulating in the community.

## Abbreviations

ACH: Acute Care Hospitals
CA-MRSA: Community-associated Methicillin-resistant *Staphylococcus aureus*
CI: Confidence Interval
DEFF: Design Effect
DNA: Deoxyribonucleic Acid
HA-MRSA: Healthcare-associated Methicillin-resistant *Staphylococcus aureus*
MRSA: Methicillin-resistant *Staphylococcus aureus*
NAG: Nasal, Axilla, Groin
NEB: New England Biolabs
NHG-DSRB: National Health Group Domain Specific Review Board
PCR: Polymerase Chain Reaction
ST: Sequence Type
WGS: Whole Genome Sequencing
